# Cyclin A and cyclin D1 as significant prognostic markers in colorectal cancer patients

**DOI:** 10.1186/1471-230X-4-22

**Published:** 2004-09-23

**Authors:** Abeer A Bahnassy, Abdel-Rahman N Zekri, Soumaya El-Houssini, Amal MR El-Shehaby, Moustafa Raafat Mahmoud, Samira Abdallah, Mostafa El-Serafi

**Affiliations:** 1 Pathology Department, National Cancer Institute, Cairo University, Cairo, Egypt; 2 Virology and Immunology Unit, Cancer Biology Department, National Cancer Institute, Cairo University, Cairo, Egypt; 3 Biochemistry Department, Kasr El-Eini School of Medicine, Cairo University, Cairo, Egypt; 4 Pathologyy Department, Kasr El-Eini School of Medicine, Cairo University, Cairo, Egypt; 5 Medical Oncology Department, National Cancer Institute, Cairo University, Cairo, Egypt

## Abstract

**Background:**

Colorectal cancer is a common cancer all over the world. Aberrations in the cell cycle checkpoints have been shown to be of prognostic significance in colorectal cancer.

**Methods:**

The expression of *cyclin D1*, *cyclin A*, *histone H3 *and *Ki-67 *was examined in 60 colorectal cancer cases for co-regulation and impact on overall survival using immunohistochemistry, southern blot and in situ hybridization techniques. Immunoreactivity was evaluated semi quantitatively by determining the staining index of the studied proteins.

**Results:**

There was a significant correlation between *cyclin D1 *gene amplification and protein overexpression (concordance = 63.6%) and between *Ki-67 *and the other studied proteins. The staining index for *Ki-67, cyclin A and D1 *was higher in large, poorly differentiated tumors. The staining index of *cyclin D1 *was significantly higher in cases with deeply invasive tumors and nodal metastasis. Overexpression of *cyclin A *and *D1 *and amplification of *cyclin D1 *were associated with reduced overall survival. Multivariate analysis shows that *cyclin D1 and A *are two independent prognostic factors in colorectal cancer patients.

**Conclusions:**

Loss of cell cycle checkpoints control is common in colorectal cancer. *Cyclin A and D1 *are superior independent indicators of poor prognosis in colorectal cancer patients. Therefore, they may help in predicting the clinical outcome of those patients on an individual basis and could be considered important therapeutic targets.

## Background

Colorectal cancer (CRC) is the third most common cancer in Western countries [1]. In Egypt, CRC has unique characteristics that differ from that reported in other countries of the western society. It was estimated that 35.6% of the Egyptian CRC cases are below 40 years of age and patients usually present with advanced stage, high grade tumors that carry more mutations [2]. This uniquely high proportion of early-onset CRC, the early and continuous exposure to hazardous environmental agents, the different mutational spectrum and the prevalent consanguinity in Egypt justify further studies [3]. It was proved that most cancers result from accumulation of genetic alterations involving certain groups of genes, the majority of which are cell cycle regulators that either stimulate or inhibit cell cycle progression [1]. Cell proliferation allows orderly progression through the cell cycle, which is governed by a number of proteins including *cyclin*s and *cyclin *dependent kinases [4,5]. The *cyclin*s belong to a superfamily of genes whose products complex with various *cyclin*-dependent kinases (*cdks*) to regulate transitions through key checkpoints of the cell cycle [6]. Abnormalities of several *cyclins *have been reported in different tumor types, implicating, in particular, *cyclin A, cyclin E *and *cyclin D *[6,7].

*Cyclin D1 *is a G1 *cyclin *that regulates the transition from G1 to S phase since its peak level and maximum activity are reached during the G1 phase of the cell cycle. Whereas *cyclin A *is regarded a regulator of the transition to mitosis since it reaches its maximum level during the S and G2 phases [8]. The mechanisms likely to activate the oncogenic properties of the *cyclins *include chromosomal translocations, gene amplification and aberrant protein overexpression [7,9].

Several studies have shown that, *histone H3 *mRNA expression can be used to identify the S phase fraction (SPF) through the in situ hybridization (ISH) technique [10,11]. The level of *histone H3 *mRNA reaches its peak during the S phase and then drops rapidly at the G2 phase [12].

In face of the increasing incidence of CRC and its peculiar pattern in the Egyptian population, the present study was conducted to assess the role of *Ki-67 *(pan-cell cycle marker), *cyclin D1 *(G1 phase marker), *histone H3 *mRNA (S phase marker), *cyclin A *(S to G2 phase marker) in CRC. The expression level of these markers was correlated to the clinicopathologic features and the overall survival of patients.

## Methods

### Tissue samples

Paraffin-embedded tumor tissues were obtained from 60 CRC patients (47 colon and 13 rectal carcinomas) that were diagnosed and treated at the National Cancer Institute, Cairo, Egypt during the period from January, 1997 to June, 2002. Clinicopathological data of the studied cases are illustrated in table 1. None of the patients received any chemotherapy or irradiation prior to surgery. Histological diagnosis of all cases was done by 2 independent pathologists according to the WHO Histological Classification. Tumors were staged according to the TNM staging system [13]. The depth of tumor invasion was classified as invasion of the mucosa including muscularis mucosa (m), invasion of the submucosa (sm), or invasion beyond the submucosa [8]. Normal colonic tissues were obtained from autopsy specimens (n = 20) and were used as a control. The actual survival rate of the patients was calculated from the date of resection to the date of death.

**Table 1:** Clinicopathological features of patients in relation to the staining index (SI) of *Ki-67*, *cyclin D1*, *cyclin A*, *histone H3*

		***SI (mean + SD)***			
		
***Variables***	***No. of cases***	***Ki-67***	***Cyclin DI***	***Cyclin A***	***Histone H3***
***Sex***					
***Male***	36	18.0 ± 6.4	6.7 ± 4.3	12.7 ± 5.7	10.7 ± 5.3
***Female***	24	20.1 ± 5.8	8.8 ± 8.4	10.0 ± 6.0	10.7 ± 5.4
***Age (years)***					
***≥50***	41	11.7 ± 6.0*	5.6 ± 5.2	10.0 ± 5.3	6.0 ± 5.0*
***<50***	19	23.8 ± 5.6	7.7 ± 6.8	13.6 ± 5.7	22.0 ± 5.2
***Tumor size (cm)***					
***<5.0***	33	12.2 ± 6.3*	5.3 ± 3.8*	11.5 ± 6.1*	10.3 ± 4.9*
***≥5.0***	27	30.1 ± 6.2	22.8 ± 7.2	28.6 ± 5.6	24.0 ± 5.6
***Histology***					
***Normal***	20	3.5 ± 2.0*	0.6 ± 0.2*	2.3 ± 1.1*	2.2 ± 0.9
***Carcinoma***	60	30.3 ± 6.2	24.9 ± 6.3	27.2 ± 5.8	10.7 ± 5.3
***GI***	15	11.7 ± 6.2	6.6 ± 4.0	10.0 ± 5.4	11.4 ± 4.9
***GII***	21	11.8 ± 5.6	8.9 ± 3.6	12.3 ± 6.5	7.8 ± 5.4
***GIII***	24	30.0 ± 4.3	22.0 ± 8.1	27.0 ± 4.9	11.5 ± 5.4
***Lymph node***					
***Negative***	33	19.5 ± 7.0	5.4 ± 5.3*	11.9 ± 6.5	12.3 ± 5.5
***Positive***	27	21.3 ± 4.9	20.6 ± 6.9	12.5 ± 5.0	14.2 ± 5.0
***Depth of invasion***					
***m, sm***	17	20.7 ± 6.7	3.1 ± 3.1*	11.9 ± 7.2	10.4 ± 5.1
***beyond sm***	43	21.9 ± 6.2	12.4 ± 6.5	12.2 ± 5.6	10.7 ± 5.4
***Stage***					
***I***	6	20.6 ± 6.7	5.7 ± 6.9	24.2 ± 6.9	11.1 ± 5.3
***II***	27	20.8 ± 6.9	5.3 ± 4.3	24.6 ± 6.0	10.4 ± 5.7
***III***	12	22.0 ± 5.4	7.7 ± 6.0	27.1 ± 5.2	10.4 ± 4.9
***IV***	15	24.7 ± 6.1	11.3 ± 9.6	27.5 ± 5.5	12.3 ± 6.2

* p. value < 0.05 (significant)

### Immunohistochemistry

Four micron sections of each normal and tumor specimen were cut onto positive-charged slides; air dried overnight, de-paraffinized in xylene, hydrated through a series of graded alcohol and washed in distilled water and 0.01 PBS (pH 7.4). Slides were then processed for IHC as described by Handa et al. [8]. using the following antibodies: *Ki-67 *(MIB-1, Dako), *cyclin A *(6E6; Novocastra, Newcastle-Upon-Tyne, UK) and *cyclin D1 *(DCS-6, Dako). A case of invasive breast cancer was used as a positive control for *Ki-67 *and *cyclin A *whereas a case of mantle cell lymphoma was used as a control for *cyclin D1*. Negative controls were obtained by replacing the primary antibody by non-immunized rabbit or mouse serum.

Brown nuclear staining was regarded as a positive result for all studied markers. The proportion of positively-stained cells and the intensity of staining were scored in tumor and normal colorectal mucosal sections at medium power (×200). The degree of positive tumor staining (percentage of positive tumor cells in the examined section) was scored from 1–6 and the staining intensity was scored from 0–6 according to the pattern of staining in the examined section. Staining index (SI) was calculated by multiplying the cellularity and staining scores as described by King et al. [14].

### In situ hybridization

All tumor samples and 5 normal controls were assessed for *histone H3 *mRNA by ISH using the commercially available 550 base fluorescein-labeled DNA probe (Dako, Carpinteria, CA) as described by Nagao et al., 1996. This probe hybridizes to the whole mRNA transcript of the human *histoneH3 *gene including the5' and 3' un-translated regions. Scoring of *histone H3 *mRNA was performed as for immunohistochemistry, however, hybridization signals were detected in the cytoplasm.

### Molecular detection of cyclin D1 gene amplification

High molecular weight DNA was extracted from paraffin-embedded tissues of the tumor and normal colorectal mucosal samples as previously described [15]. The proportion of neoplastic and normal cells was determined in each tumor sample by examining hematoxylin and eosin-stained slides obtained from the edge of the specimen used for DNA extraction. Tumor samples were evaluated for amplification of *cyclin D1 *if more than 75% of the examined sections were formed of neoplastic cells. Accordingly, 50 cases were eligible for the analysis. Ten micrograms of the extracted DNA was digested with *Eco*R1. DNA from selected cases was also digested with *Bgl*II and *Hind*III. Samples were separated on 0.8% agarose gels and transferred to Hybond-N membranes (Amersham Int., Amersham, UK). The membranes were hybridized with 50% formamide, 5 × SSC, 5 × Denhardt's, 500 μg/ml denatured salmon sperm DNA, 10% dextran sulphate and 10^6 ^cpm/ml of ^32^P-labeled *PRAD-1 *probe for 24 h. Membranes were washed with 2 × SSC, 0.1% SDS at room temperature for 30 min followed by 2 × SSC, 0.1% SDS at 60°C for 30 min and 0.1 × SSC, 0.1% SDS at 60°C for 1 h. Filters were autoradiographed using an intensifying screen at -70°C for 24–72 h. After being stripped free of the *PRAD-1 *probe, the same blots were hybridized with ^32^P-labeled *B-actin *probe to normalize against possible variations in the loading or transfer of DNA. The autoradiograms were analyzed using a densitometer. Intensities of *PRAD-1/cyclin D1 *were normalized to the *β-actin *control bands. The degree of amplification was calculated from these normalized values. Amplification was considered when the signal of the tumor band was ≥2-fold the value of the matched normal mucosa [16].

### Statistical analysis

The Mann-Whitney non-parametric test was used to compare the SIs of pairs of subjects whereas the Kruskal-wallis was used for categorial data. Correlation between indices was performed using a simple linear regression test. The Kaplan-Meier method was used to create survival curves which were analyzed by the log-rank test. The impact of different variables on survival was determined using the Cox proportional hazards model. *p*. values less than 0.05 were considered significant.

## Results

**Figure 1:**
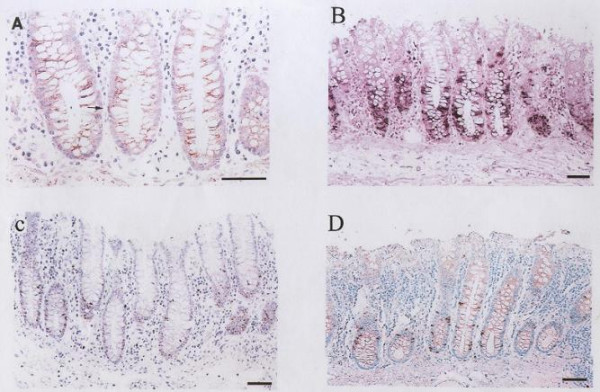
Normal colonic mucosa showing positive nuclear immunostaining for: (a) *cyclin D1*, (b) ISH of *histone H3 *mRNA, (c) *Ki-67 *and (d) *cyclin A*

**Figure 2:**
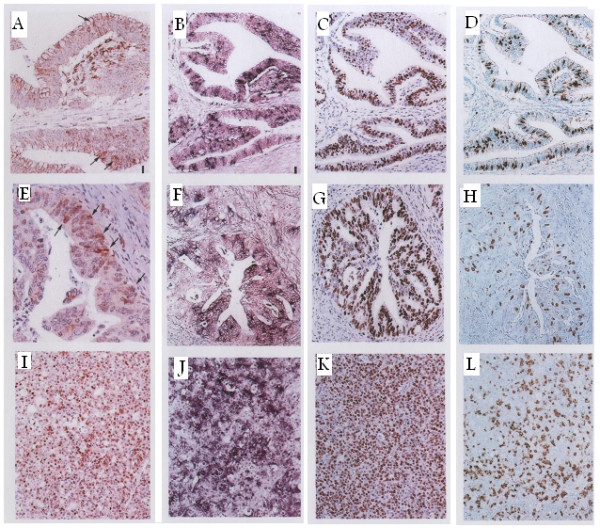
A case of well differentiated adenocarcinoma with positive immunostaining for: (a) *cyclin D1*, (b) *histone H3 *mRNA, (c) *Ki-67*, and (d) *cyclin A*. Another case of moderately differentiated denocarcinoma with positive immunostaining for: (e) *c*yclin D1, (f) *histone H3 *mRNA, (g) *Ki-67*, and (h) *cyclin A*. A case of poorly differentiated adenocarcinoma with diffuse staining for: (i) *cyclin D1*, (j) ISH of *histone H3 *mRNA, (k) *Ki-67 *and (l) *cyclin A*.

The results of IHC are illustrated in figures 1 and 2. In general, the staining index (SIs) of all studied markers was higher in carcinomas than in normal colonic mucosal samples (*p *= 0.0001). Normal colorectal mucosa revealed positive imunostaining for *Ki-67 *in the lower half of the crypts only. A heterogeneous staining pattern was detected in the neoplastic cells of well and moderately-differentiated adenocarcinomas whereas a diffuse homogeneous staining pattern was detected in poorly-differentiated carcinomas. The SI ranged from 10–40.2 (mean: 24.6 ± 6.5).

Immunostaining for *cyclin D1 *was predominantly nuclear but cytoplasmic staining was detected in some cases. However, unless a nuclear staining was also detected, cases with cytoplasmic staining were considered negative. Normal colorectal mucosal samples were almost negative for *cyclin D1 *whereas 41 out of the 60 (68.3%) CRC cases were positive. Marked heterogeneity was observed in well- and moderately-differentiated adenocarcinomas even within the same tumor. Poorly-differentiated carcinomas revealed a diffuse staining pattern with more darkly-stained nuclei. The SI ranged from 0.5–28.6 (mean: 9.3 ± 4.2).

Positive nuclear staining for *cyclin A *was detected in 80% (48/60) of CRC cases and in all non-neoplastic control samples. Positively-stained nuclei were confined to the lower half of the crypts in normal colonic mucosa and diffusely-dispersed in carcinomas. The SI ranged from 3.3–30.2 (mean: 15.1 ± 6.6).

*Histone H3 *mRNA was intensely expressed in the cytoplasm of all examined samples either neoplastic or non-neoplastic. The distribution of *histone H3 *mRNA was similar to that of *cyclin A *and *Ki-67 *however, the proportion of *histone H3 *mRNA positive cells was less than that of *Ki-67*. The SI ranged from 1.8–24.2 (mean: 12.4 ± 5.3).

**Figure 3:**
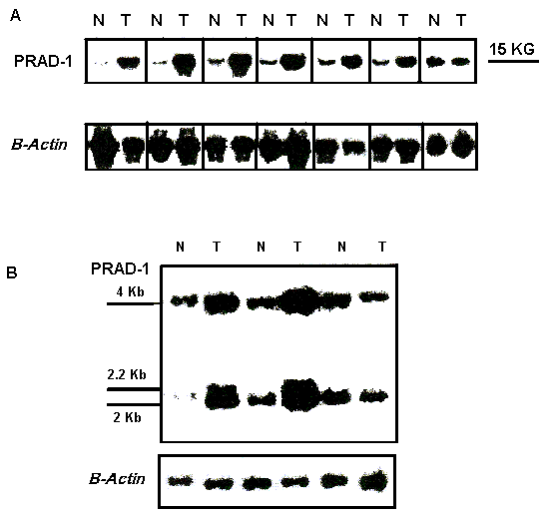
**A: **Southern blot analysis of normal mucosa (N) and their seven corresponding cases of colonic adenocarcinomas (T1–T7), cases No. 1, 2, 4, and 5 are poorly differentiated whereas cases No. 3, 6, and 7 are moderately differentiated. Genomic DNA was digested with *Bgl*II, fractionated by electrophoresis in agarose gel, transferred onto membranes and hybridized with *PRAD1 *and *β-actin*. Tumors number 1–6 (Lanes 1–6) show different degrees of *PRAD1/cyclin D1 *amplification, tumor number 7 (lane 7) was not amplified. **B**: Southern blot analysis of 3 cases of adenocarcinomas (T) and matched normal colonic mucosa (N). Genomic DNA was digested with *Eco*RI, fractionated by electrophoresis in agarose gel, transferred onto membranes and hybridized with *PRAD1 *and *β-actin *probes for loading control. The identification of the 3 tumors is the same as in Fig. 3A with amplification of *PRAD1/cyclin D1 *in tumors number 4, 5 (Lanes 1, 2) but not 7 (Lane 3).

The *PRAD-1 *probe detected 3 *Eco*RI fragments of 4.0, 2.2 and 2.0 and 1 *Bgl*II fragment of 15 Kb. *PRAD-1/cyclin D1 *gene amplification was detected in 22/50 (44%) cases analyzed. The degree of amplification was heterogeneous with 2–10 fold increase when compared to normal mucosal samples (Figure 3). Amplification was confirmed by other restriction enzymes.

### Correlations

There was a significant correlation between *cyclin D1 *gene amplification and protein overexpression. Out of the 22 cases that showed amplification 14 showed protein overexpression (concordance = 63.6%).

**Figure 4:**
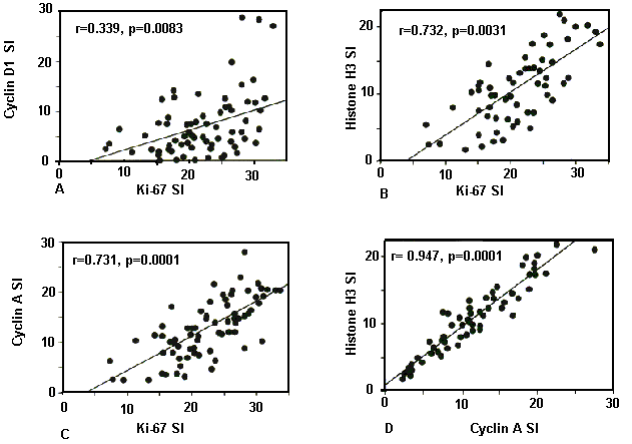
Correlation between the staining intensity of (a) *Ki-67 vs. cyclin D1*, (b) *Ki-67 vs. histone H3*, (c) *Ki-67 vs. cyclin A *and (d) *cyclin A vs. histone H3 *mRNA expression.

Linear regression analysis of SIs revealed a significant correlation between *Ki-67 *and *cyclin D1, cyclin A, histone H3 *as well as between the SIs of *cyclin A *and *histone H3 *(*p *= 0.008, 0.0001, and 0.0001 respectively) (Figure 4). There was a significant relationship between the SI of both *Ki-67 *and *cyclin A *and the degree of differentiation of tumors as well as the size of the tumor (*p *< 0.001 and *p *< 0.01 respectively). In addition, SI of *Ki-67 *and *histone H3 *were higher in patients <50 years than in those ≥50 years (*p *< 0.05) (table 1).

In addition table 2 shows a significant relationship between high *cyclin D1 *SI and large, poorly-differentiated tumors, carcinomas with positive lymph node metastasis and deeply-invasive carcinomas (*p *< 0.05, *p *< 0.001, *p *< 0.05 and *p *< 0.05 respectively). Whereas *cyclin D1 *gene amplification was significantly associated with an advanced disease stage since amplification was detected in 10/15 (66.7%) of stage IV tumors compared to 12/45 (26.7%) of stage I-III tumors (*p *= 0.002). Similarly, DNA amplification was detected in 60.5% (26/43) of the carcinomas with extensive local invasion (beyond sm) but only in 23.5% (4/17) of the carcinomas with limited invasion (m, sm) (*p *= 0.001). A significant correlation was also present between *cyclin D1 *gene amplification and the presence of lymph node metastasis (*p *= 0.008) as well as between the SI of *histone H3*, the size of the tumor and the patient's age (*p *< 0.05, *p *< 0.001 respectively). The SI was higher in tumors >5 cm in diameter and in patients <50 years.

**Table 2:** The relation between *cyclin D1 *overexpression vs *cyclin D1 *amplification and clinicopathological prognostic markers.

***Variables***	***No. of cases***	***Cyclin DI overexpression***	***Cyclin D1 Amplification***
***Tumor size (cm)***			
***<5.0***	33	5.3 ± 3.8*	13/33
***≥5.0***	27	22.8 ± 7.2 **p **<***0.05***	9/27 p <*0.236*
***Histology***			
***GI***	15	6.6 ± 4.0	7/15
***GII***	21	8.9 ± 3.6	8/21
***GIII***	24	22.0 ± 8.1 **p **<***0.001***	7/24 **p **<***0.075***
***Lymph node***			
***Negative***	33	5.4 ± 5.3*	6/33 (18.2%)
***Positive***	27	20.6 ± 6.9 **p **<***0.05***	16/27 (59.3%) **p **<***0.008***
***Depth of invasion***			
***m, sm***	17	3.1 ± 3.1*	4/17 (23.5%)
***beyond sm***	43	12.4 ± 6.5 **p **<***0.05***	26/43 (60.5%) **p **<***0.001***
***Stage***			
***early***	45	5.5 ± 10.1	12/45 (26.7%)
***late***	15	11.3 ± 9.6 P = *0.175*	10/15 (66.7%) **p **<***0.002***

### Survival analysis

**Figure 5:**
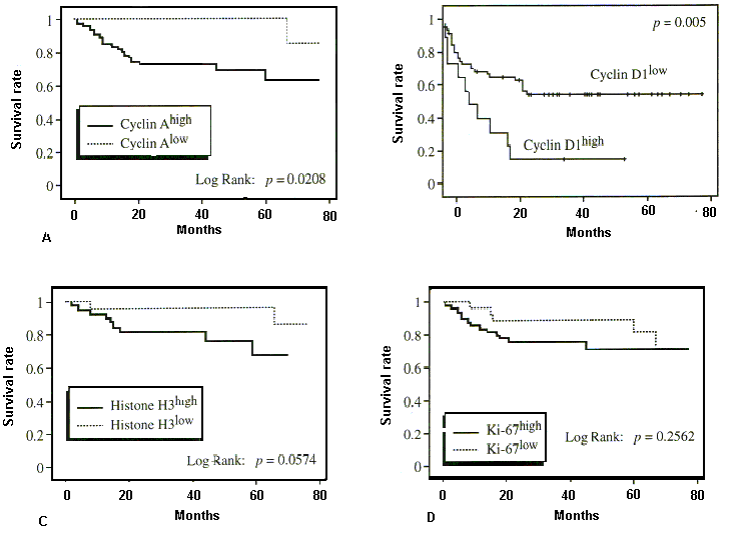
Kaplan-Meier survival curves for colorectal carcinoma. Overall survival is significantly lower in patients with (a) *cyclin A *and (b) *cyclin D1 *overexpression. Patients with high SI for *histone H3 *mRNA have poorer prognosis but this was not statistically significant (c). No significant difference was present between patients with high *Ki-67 *SI and those with low *Ki-67 *SI (d).

The mean follow-up period for all patients was 30 months (range: 1–66 months). Eighteen of 60 patients had already died by the time the study was completed. We defined the cutoff level for overexpression of each cell cycle marker at the point that showed the maximum difference of survival rate between the 2 groups separated by that point. Cox regression analysis revealed that *cyclin A *overexpression (our definition: SI ≥ 10.5), *cyclin D1 *overexpression (our definition: SI ≥ 6.1), poorly differentiated histology, lymph node metastasis, TNM stage, tumor size and depth of invasion were all significant prognostic variables for survival (Table 3). The Kaplan-Meier survival curves for the subgroups of patients who are subdivided according to each marker's status are shown in Figure 5. Patient with tumors that showed *Ki-67 *overexpression (our definition: SI ≥ 11.5) and *histone H3 *overexpression (our definition: SI ≥ 8.2) tended to have poor prognosis but this did not reach a statistically significant level, however the overall survival was significantly lower in patient with *cyclin A *and *cyclin D1 *overexpression. Cox multivariate regression analysis revealed that lymph node metastasis, *cyclin A *and *cyclin D1 *overexpression were independent negative prognostic factors after adjustment for the depth of tumor invasion, age and sex of the patient (Table 4).

**Table 3:** Uunivariate analysis of the relationship between survival and the tested markers

***PredictiveVariables***	***Median Survival***	***HR***	***CI***	***P***
***Ki-67***				
<11.5	36			
≥11.5	32	1.826	0.636 – 5.243	0.26
***Cyclin D1***				
<6.1	35			
≥6.1	18	7.246	1.007 – 45.150	0.03*
***Histone H3***				
<8.2	35			
≥8.2	29	4.639	0.854 – 25.196	0.07
***Cyclin A***				
<10.5	35			
≥10.5	15	7.820	1.017 – 60.122	0.02*
***Histological grade***				
***Low***	38			
***High***	10	7.331	2.696 – 19.940	0.0001*
***Lymph node***				
***Negative***	38			
***Positive***	15	6.826	1.973 – 23.621	0.002*
***Stage***				
***I, II, III***	38			
***IV***	12	6.378	1.842 – 22.083	0.001*
***Tumor size (cm)***				
<5.0	35			
≥5.0	13	4.835	1.386 – 16.868	0.01*
***Depth of invasion***				
T1, T2	36			
T3, T4	20	7.759	1.024 – 58.789	0.04*
***Age (years)***				
<50	38			
≥50	28	2.802	0.988 – 7.943	0.0526
***Sex***				
***Male***	38			
***Female***	36	0.696	00.274 – 1.766	0.4449

* p. value < 0.05 (significant)

HR: Hazard Ratio

CI: 95% confidence Interval

**Table 4:** Multivariate analysis ofthe relationship between survival and thetested markers

***PredictiveVariables***	***HR***	***CI***	***P***
***Cyclin D1***	10.864	1.055 – 86.250	0.03*
(baseline < 6.1)	-	-	-
***Cyclin A***	13.886	1.012 – 190.579	0.0490*
(baseline < 10.5)	-	-	-
***Positive Lymph node metastasis***	3.921	1.057 – 14.472	0.0410*
***Stage IV***	3.411	1.048 – 12.083	0.03*
***Depth of invasion***			
T3, T4	5.408	0.449 – 65.080	0.1836
***Age (years)***			
≥50	1.996	0.678 – 5.878	0.2310
***Sex***	0.910	0.315 – 2.358	0.8453

p. value < 0.05 (significant)

HR: Hazard Ratio

CI: 95% confidence Interval

## Discussion

The proliferative activity of CRC cells has been investigated in several studies either by immunohistochemical determination of cell proliferation index using antibodies to some types of *cyclins *or by flowcytometric determination of the SPF of the cell cycle [8]. Although Leach et al. [17] did not find *cyclin D1 *gene amplification in a panel of 47 CRC cell lines; its protein was overexpressed in about 30% of CRC cases that were included in the studies of Bartakova et al. [6] and Arber et al. [18]. In the former study [6]*cyclin D1 *was aberrantly accumulated in a significant subset of human CRC cases and the cell lines derived from these cases were dependent on *cyclin *in their cell cycle progression. In the second study [18], overexpression of *cyclin D1 *was detected in 30% of adenomatous polyps indicating that overexpression is a relatively early event in colon carcinogenesis which is possibly responsible for the pathological changes in the mucosa preceding neoplastic transformation. More recently, Holland et al. [19], Pasz-Walczak et al. [20] and Utsunomiya et al. [21] reported up-regulation of *cyclin D1 *in 58.7%, 100% and 43% of their studied cases respectively.

In the present study, up-regulation of *cyclin D1 *was detected in 68.3% of the cases. The SI was significantly higher in carcinomas than in normal colorectal mucosa and in poorly-differentiated adenocarcinomas it was approximately twice that of other histological types. Amplification and/or overexpression of *cyclin D1 *significantly correlated with deeply invasive tumors and positive lymph node metastasis. Our results in this regards are consistent with previous studies [8,22]. In 2001, Holland et al. [19]. demonstrated that deregulation of *cyclin D1 *and *p21*^*waf *^proteins are important in colorectal tumorigenesis and have implications for patient prognosis. Similarly McKay et al. [23] found that *cyclin D1 *was the only protein in their panel (*cyclin D1, p53, p16, Rb-1, PCNA and p27*) that correlated with improved outcome in CRC patients. However, few studies failed to detect any correlation between *cyclin D1 *overexpression and the clinicopathological factors in CRC [6,18]. This controversy in results could partially be explained by the difference in the sampling of studied cases. The present study included 24 cases of poorly differentiated adenocarcinoma, which is not common in other studies of CRC in western countries. This was possible because the majority of CRC cases diagnosed in Egypt are of high histological grade [3]. The correlation between *cyclin D1 *overexpression and the high histological grade was also reported in other tumor types including non-small cell lung carcinomas [24] and squamous cell carcinomas of the larynx [16]. Another possible explanation for the observed controversy in the results of different studies is the detection method used.

In the present work, overexpression of *cyclin D1 *was more common than gene amplification of the *PRAD-1/cyclin D1 *gene with a 63.6% concordance. This was similarly reported by Bartakova et al. [6] who mentioned that there is a subset of CRC cases in which *cyclin D1 *is overexpressed without *PRAD-1/cyclin D1 *gene amplification. Consistent with this hypothesis are reports of elevated *cyclin D1 *mRNA levels and immunohistochemically detectable accumulation of the protein in over one third of breast cancer cases at a frequency significantly higher than that deduced from DNA amplification studies [9,25]. These data imply that mechanisms other than gene amplification can also lead to deregulation and accumulation of *cyclin D1 *in solid tumors.

So far, several studies were done to reveal the prognostic significance of *cyclin D1 *overexpression in various carcinomas, including CRC [22]. However, these studies yielded conflicting results which could be attributed to organ heterogeneity. In our study, patients with tumors that exhibited *cyclin D1 *overexpression tended to have poor prognosis.

It was reported that, patients with *cyclin A *positive carcinomas had significantly shorter median survival times. Handa et al. [8] were able to detect *cyclin A *overexpression in 77% of their CRC cases. They also demonstrated that, *cylcin A *could be used as a prognostic factor of CRC. More recently, Habermann et al. [26] studied cases of ulcerative colitis with and without an associated adenocarcinoma for the presence of *cyclin A *overexpression. They found that, *cyclin A *overexpression was higher in cases of ulcerative colitis with adenocarcinomas than in those without adenocarcinomas. Consequently, they concluded that, *cyclin A *could be used for monitoring ulcerative colitis patients and for the early detection of an emerging carcinoma in this high risk group of patients.

In our study, *cyclin A *was detected in 80% of the patients and Cox regression analysis showed that it could be used as a prognostic marker in CRC in addition to *cyclin D1*.

It would have been useful if we assessed the expression level of *cyclin A *by another technique (DNA amplification). This would have added more information regarding the gene status on one hand and confirmed the results of IHC on the other hand. Unfortunately, this was not possible because in most of the cases included in the present work, the extracted DNA was not sufficient to study *cyclin amplification *after the assessment of *cyclin D1*.

In 1996, Nagao et al. [11] reported that *histone H3 *labeling index significantly correlated with ki-67 immunostaining and was high in poorly differentiated human hepatocellular carcinoma. This was similarly reported in the present work since we found a significant correlation between the SI of *histone H3 *and *Ki-67*. However, no statistically significant correlation was found between *histone H3 *SI and any of the studied clinicopathological factors.

Although *Ki-67 *immunostaining reflects the proliferative activity of CRC, it has not been recognized as a significant prognostic factor in this type of tumors [27,28]. However, Suzuki at al. [29] found a significant correlation between *Ki-67 *labeling index and local invasion of CRC. In the present study there was a significant relationship between the SI of *Ki-67*, tumor size and grade. However, Kaplan-Meier survival curves showed no significant difference in survival rates between patients with- and without overexpression of *Ki-67*.

## Conclusions

Our results demonstrate that *cyclin D1, cyclin A, histone H3 *and *Ki-67 *are overexpressed in a subset of CRC, however only *cyclin D1 *and *cyclin A *overexpression correlates with poor differentiation and tumor progression. This indicates the superiority of *cyclin A *and *cyclin D1 *as indicators of poor prognosis compared to *Ki-67 *and *histone H3 *mRNA in CRC. *Cyclin A and D1 *could therefore be considered significant, independent prognostic factors in CRC patients. These findings are especially important in stage II patients since 25–30% of those patients have poor prognosis in spite of being node-negative. However, the standard clinicopathologic prognostic factors can not identify this subset accurately and therefore; there is a great demand for more accurate, individually-based, biological prognostic parameters that help in detecting this high risk group of patients who can benefit from an adjuvant therapy. If the findings of the present study are confirmed in a larger study, evaluation of *cyclin A and D1 *may be applicable to clinical management of CRC, allowing the identification of patients with poor prognosis.

## Competing interests

The author(s) declare that they have no competing interests.

## List of abbreviations

CRC – Colorectal cancer

OS – overall survival

SI – staining index

SPF – S phase fraction

ISH – in situ hybridization

m – muscularis mucosa

sm – invasion of the sub mucosa

## Authors' contributions

BA and ZA-R carried out the molecular genetic studies, designed, coordinated the study and drafted the manuscript. BA and El-HS carried out all the histopathological and immunohistochemical studies. El-SA participated in molecular genetic studies and drafted the manuscript. MM coordinated the study. El-SM carried out all the patient clinical data. All authors read and approved the final manuscript

## Pre-publication history

The pre-publication history for this paper can be accessed here:


